# MG53 Inhibits Necroptosis Through Ubiquitination-Dependent RIPK1 Degradation for Cardiac Protection Following Ischemia/Reperfusion Injury

**DOI:** 10.3389/fcvm.2022.868632

**Published:** 2022-05-31

**Authors:** Qiang Wang, Ki Ho Park, Bingchuan Geng, Peng Chen, Chunlin Yang, Qiwei Jiang, Frank Yi, Tao Tan, Xinyu Zhou, Zehua Bian, Jianjie Ma, Hua Zhu

**Affiliations:** Department of Surgery, Davis Heart and Lung Research Institute, The Ohio State University, Columbus, OH, United States

**Keywords:** redox, MG53, RIPK1, necroptosis, I/R injury, ubiquitination

## Abstract

**Rationale:**

While reactive oxygen species (ROS) has been recognized as one of the main causes of cardiac injury following myocardial infarction, the clinical application of antioxidants has shown limited effects on protecting hearts against ischemia–reperfusion (I/R) injury. Thus, the precise role of ROS following cardiac injury remains to be fully elucidated.

**Objective:**

We investigated the role of mitsugumin 53 (MG53) in regulating necroptosis following I/R injury to the hearts and the involvement of ROS in MG53-mediated cardioprotection.

**Methods and Results:**

Antioxidants were used to test the role of ROS in MG53-mediated cardioprotection in the mouse model of I/R injury and induced human pluripotent stem cells (hiPSCs)-derived cardiomyocytes subjected to hypoxia or re-oxygenation (H/R) injury. Western blotting and co-immunoprecipitation were used to identify potential cell death pathways that MG53 was involved in. CRISPR/Cas 9-mediated genome editing and mutagenesis assays were performed to further identify specific interaction amino acids between MG53 and its ubiquitin E3 ligase substrate. We found that MG53 could protect myocardial injury *via* inhibiting the necroptosis pathway. Upon injury, the generation of ROS in the infarct zone of the hearts promoted interaction between MG53 and receptor-interacting protein kinase 1 (RIPK1). As an E3 ubiquitin ligase, MG53 added multiple ubiquitin chains to RIPK1 at the sites of K316, K604, and K627 for proteasome-mediated RIPK1 degradation and inhibited necroptosis. The application of N-acetyl cysteine (NAC) disrupted the interaction between MG53 and RIPK1 and abolished MG53-mediated cardioprotective effects.

**Conclusions:**

Taken together, this study provided a molecular mechanism of a potential beneficial role of ROS following acute myocardial infarction. Thus, fine-tuning ROS levels might be critical for cardioprotection.

## Graphical Abstract

The schematic figure shows MG53 mediated RIPK1 degradation is enhanced by ROS generated in infarct hearts. Thus, fine tune of ROS level is critical for post-MI cardioprotection. 
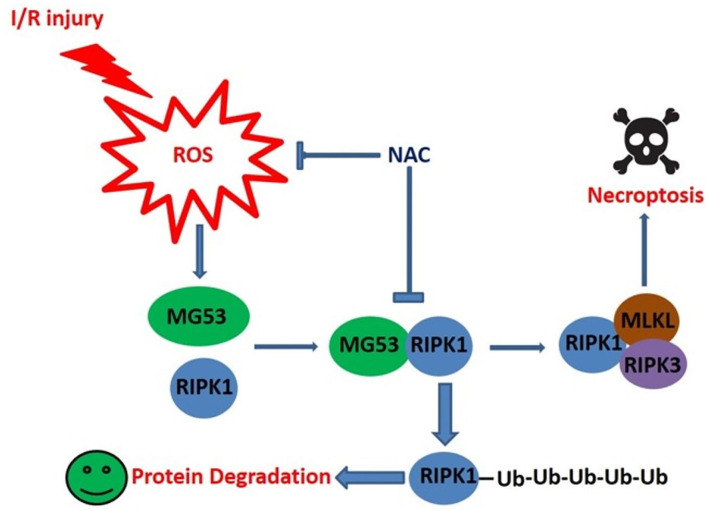

## Highlights

- Post-cardiac ischemic injury induces reactive oxygen species (ROS) production.
- ROS promotes interaction between MG53 and RIPK1 in infarct myocardium.
- MG53 serves as a ubiquitin E3 ligase of RIPK1 for its degradation.
- MG53 inhibits cardiomyocyte necroptosis and protects against cardiac injury.
- Fine-tune cardiac redox status, rather than scavenging total ROS, is critical for cardioprotection.

## Introduction

Ischemic heart disease is a major threat to public health in the US and around the world (1). Acute myocardial infarction (MI) results in extensive damage to the myocardium that causes loss of cardiac cells and subsequent development of heart failure (HF). While extensive studies have been carried out in the field of MI, the detailed molecular and cellular events that lead to this devastating cardiac syndrome are still largely unknown. Thus, it is highly desired if one can elucidate the molecular mechanisms underlying cardiac cell death.

Over the past decades, emerging studies have suggested the existence and significance of necroptosis in the development of HF (2–4). Unlike necrosis, necroptosis is highly programed, which could be a potential druggable target for the treatment of cardiac diseases. However, the detailed molecular regulation of necroptosis in cardiovascular diseases is still largely unknown.

MG53 is encoded by the *TRIM72* gene, which belongs to the family of tripartite motif-containing (TRIM) proteins and is predominantly expressed in the skeletal muscle and the heart in mice (1, 5, 6). Previous studies have demonstrated that MG53 is an indispensable component for plasma membrane repair machinery (6). Studies from our and other groups have demonstrated that either virus-mediated delivery of the MG53 gene or administration of recombinant human MG53 (rhMG53) protein could protect against injuries in many tissues, such as the heart (7–10), the skeletal muscle (11–14), the kidney (15), the liver (16), the brain (17), the lung (18), the cornea (19), and skin (14). These profound effects of MG53 have inspired us to explore novel functions of MG53 in tissue protection.

In the present study, we first studied the potential synergistic effects of rhMG53 and N-acetyl cysteine (NAC) on cardiac protection in an I/R-induced murine cardiac injury model. The rationale was that rhMG53 could protect against acute cell membrane injury and that NAC could inhibit reactive oxygen species (ROS) production following I/R injury. To our surprise, the combination of rhMG53 and NAC treatment exacerbated the cardiac injury as compared to rhMG53 treatment alone. The immunoprecipitation analysis revealed that NAC treatment disrupted the interaction between MG53 and receptor-interacting protein kinase 1 (RIPK1), a key factor of necroptosis. Further biochemical and molecular biology studies demonstrated that MG53 could serve as a ubiquitin E3 ligase for RIPK1. MG53-mediated degradation of RIPK1 could inhibit necroptosis and protect the myocardium against I/R injury. Thus, fine-tune redox status, rather than ROS scavenging, might be critical for cardiac protection.

## Methods and Materials

### Animals

The *mg53-/-* and wild-type littermate mice were maintained in a C57BL/6-129sv mixture background. tPA-MG53 mouse line was kept in the C57BL/6 background. Due to the differences in genetic backgrounds, wild-type littermate mice were used for *mg53-/-* and tPA-MG53 mice. tPA-MG53 transgenic mice were generated by the addition of tPA secretion signaling peptide on the N-terminus of the MG53 protein sequence (Cyagen Biosciences. Santa Clara, CA, USA). The produced tPA-MG53 is secreted into the bloodstream to achieve a high level of circulating MG53. The tPA-MG53 mice were used in our previous studies (20, 21). Mice were kept under 12-h light/dark cycle within a temperature-controlled environment and had free access to food and water. Only male mice were used in the surgery. The experimental protocol was approved by the Institutional Animal Care and Use Committee at The Ohio State University Wexner Medical Center.

### I/R Injury in Mice

Adult wild-type, *mg53-/-*, and tPA-MG53 mice were used in the heart I/R injury surgery. Mice were anesthetized by the isoflurane (Piramal Enterprises Limited) under a small animal respirator (Harvard apparatus). A middle sternotomy was performed and the left anterior descending coronary artery was ligated by an 8-0 silk suture. Acute I/R injury was induced by suture ligation for 40 min and followed by reperfusion. Then, 2 h after reperfusion, the blood samples were collected for creatine kinase (CK) measurement. The 6-h reperfusion hearts were used for TTC staining to measure infarction size and tissue collection for western blotting analysis. For NAC treatment, low (50 mg/kg) and high doses (250 mg/kg) of NAC were administrated by intraperitoneal (IP) injection 30 min before ischemia and right after reperfusion.

For CK detection, blood samples were collected and mixed with heparin (Sigma) and centrifuged for 5 min at 3,000 rpm to obtain plasma. The CK measurement assay used a kit from Sekisui Diagnostics Limited following the company's protocol.

For heart infarct size measurement, mice were heparinized and excised the heart from the chest. The site of occlusion was found and used the snare to close the coronary artery again. The heart was perfused with phosphate-buffered solution (PBS) first and then used 10% phthalo blue (Utrecht Mfg Corp.) through the ascending aorta to visualize the area at risk. The heart was placed into the refrigerator at −80°C for 10 min and was cut into 5 slices. Heart slices were incubated for 15 min in PBS containing 1% 2, 3, 5-triphenyl tetrazolium chloride (Sigma) to visualize the unstained infarcted area. Infarct area, area at risk, and left ventricular area were calculated by the ImageJ software.

### Echocardiography and Histology

Mice were anesthetized by isoflurane, and echocardiography was performed according to the manufacturer's protocol. After 4 weeks of I/R surgery (1-h ischemia), hearts were fixed in 4% paraformaldehyde, embedded, and sectioned. Heart fibrosis was stained by using the Picrosirius red stain kit (Polysciences, Inc.) and quantified by ImageJ software.

### Cell Culture, Plasmid Transfection, and Drug Treatment

HEK293 cells were cultured in high glucose DMEM (Gibco) supplemented with 10% fetal bovine serum (FBS) (Gibco) and 1% penicillin–streptomycin (Gibco). HA-RIPK1 and point mutant plasmids of HA-RIPK1 (K3R, K115R, K163R, K316R, K571R, K604R, and K627R) were gifts from Jaewhan Song (obtained from Addgene). MG53 C14A and HA-RIPK1 double mutant plasmids were generated by site-directed mutagenesis (New England Biolabs). HA-RIPK1 triple mutant plasmid was generated by the Mutagenex Company. The day before plasmids transfection, HEK293 cells will passage to new plates. After transfection, the cells will be collected for further experiments.

Human-induced pluripotent stem cells (hiPSC) were purchased from Gibco (A18945) and kept in the Essential 8 basal medium (Gibco). The cardiomyocyte differentiation kit (Gibco) was used for cardiomyocyte differentiation following our published protocol (22). After cardiomyocyte differentiation, 0.5% oxygen in nitrogen gas (99.5%) was used for hypoxia and the re-oxygen (H/R) treatment. The re-oxygenation condition was the same as the culture condition (95% air and 5% CO_2_). Recombinant protein MG53 was added to the medium before hypoxia treatment.

CRISPR/Cas 9-mediated *mg53-/-* cell and wild-type parental cells were also used for H/R treatment.

### Western Blotting and Immunoprecipitation

The heart tissues and cells were lysed using a lysis buffer composed of 50 mM Tris-HCl (pH7.5), 150 mM NaCl, 10% glycerol, 1% Triton X-100, 1 mM ethylenediaminetetraacetic acid (EDTA), and protease inhibitors (Sigma). The lysates were incubated in the following antibodies: anti-RIPK1 (610459, BD Bioscience) (1:1,000 dilution), anti-RIPK3 (ab56164, Abcam and 95702S, CST) (1:1,000 dilution), anti-caspase 8 (3B10 and 12F5, Enzo) (1:1,000 dilution), anti-cleave caspase 3 (9664S, CST) (1:1,000 dilution), anti-MG53 (made in rabbit) (1:5,000 dilution), anti-LC3A/B (12741S, CST) (1:1,000 dilution), anti-Ub (3936S, CST) (1:1,000 dilution), anti-His (2365S, CST) (1:1,000 dilution), anti-actinin (A7811, CST) (1:1,000 dilution), anti-β-actin (A5441, Sigma) (1:5,000 dilution), anti-HA (26183, Pierce and 3724S, CST) (1:1,000 dilution), and anti-flag (F1804, Sigma) (1:1,000 dilution). The relative band intensity was acquired using the Quantity One software.

Before adding the cell lysate or tissue lysate, the Dynabeads Protein G (Invitrogen) was incubated with the antibody for 1 h. Then, cell or tissue protein was added and incubated in a cold room overnight. The next day, the pulldown results were detected using a western blotting assay. The following antibodies were used in the immunoprecipitation: anti-RIPK1 (610459, BD Bioscience) (1:500 dilution), anti-MG53 (made in rabbit) (1:2,000 dilution), and anti-HA (Pierce, 26183) (1:500 dilution) for pulldown. Anti-RIPK1 (610459, BD Bioscience) (1:1,000 dilution), anti-RIPK3 (ab56164, Abcam and 95702S, CST) (1:1,000 dilution), anti-MG53 (made in rabbit) (1:5,000 dilution), anti-anti-Ub (3936S, CST) (1:1,000 dilution), anti-actinin (A7811, CST) (1:1,000 dilution), and anti-β-actin (A5441, Sigma) (1:5,000 dilution) were to determine extraction protein expression levels.

### Immunofluorescence

After the hiPSC was differentiated for 10 days to the state of beating cardiomyocyte, cells were placed into the nitrogen incubator for hypoxia treatment. Then, the cells went back to the re-oxygen environment and changed the medium with 0.5 μg/ml propidium iodide (PI) and 50 ug/ml human MG53 recombinant protein for treatment or not. Immunocytochemical images were acquired by a Zeiss immunofluorescence microscope system.

### Recombinant Protein Production and Purification

His-MG53 and His-MG53 C14A protein were expressed in BL21 cells and purified by the His-antibody beads. The recombinant protein-binding beads were washed three times and then eluted to collect the protein solution. After collecting the recombinant proteins, Coomassie blue staining and western blotting were used to determine protein expression.

### *In vitro* Ubiquitination Assay

The protocol of *in vitro* ubiquitination was reported and modified as described previously. About 2 μg His-RIPK1 (TP760433, OriGene Technologies, Inc), 100 ng E1 (UBE1; E-305; Boston Biochem), 250 ng E2 (UbcH2; E2-607; Boston Biochem), 5 μg ubiquitin(Sigma), and 2 mM ATP(Sigma) were incubated in the absence or presence of purified Flag-MG53 and Flag-MG53 C14A in 40 mM Tris-HCl (pH7.6), 50 mM NaCl, and 1 mM dithiothreitol for 5 h. After the incubation, the samples were added two times of loading buffer and boiled. Using anti-His antibody to analyze the samples by western blotting.

### Statistical Analysis

Data are presented as means ± SEM. Statistical analysis was performed with GraphPad Prism 8. Comparison of means was performed using the ANOVA followed by adequate *post-hoc* tests.

## Results

### MG53 Protects Against I/R-Induced Cardiac Injury

Previous studies have shown the role of MG53 in cardiac protection. In the current study, we confirmed MG53's cardiac protection effects using *mg53-/-* mice and their wild-type (*wt*) littermate controls. Consistent with the previous studies (8–10), *mg53-/-* hearts are susceptible to I/R injury as evidenced by significantly larger myocardial infarct area as compared to that of wt hearts (33.7% in *mg53-/-* vs. 16.3% in *wt, p* = 0.0023), whereas the area at risk is comparable between two mouse lines (40.4% in *mg53-/-* vs. 46.3% in *wt*) (Supplementary Figures S1A–C). Furthermore, we found that genetic ablation of MG53 led to a significant increase of creatine kinase release following cardiac I/R injury as compared to *wt* controls (Supplementary Figure S1D). Next, we evaluated chronic cardiac pathologies 4 weeks after I/R injury. We found that the loss of MG53 significantly enhanced fibrotic cardiac remodeling (13.8% in *mg53-/-* vs. 6.4% in *wt, p* = 0.003, Supplementary Figure S1E) and decreased cardiac contractile dysfunctions as evidenced by echocardiogram measurement of both ejection fraction and fractional shortening (Supplementary Figures S1F,G).

We also utilized a transgenic mouse line in our study. tPA-MG53 transgenic mice were generated by conjugating a secretory signaling peptide sequence (tPA) to the N-terminus of MG53 transgene to achieve a high expression level of MG53 in blood circulation (20, 23), which serves as a gain-of-function animal model. Consistent with our previous studies in I/R-induced brain (23) and cardiotoxin-induced skeletal muscle injury (20), tPA-MG53 mice showed resistance to I/R-induced cardiac injury, as evidenced by reduced infarct size (30.8% in wt vs. 14.8% in tPA-MG53 mice, *p* = 0.0025) (Supplementary Figures S2A–C). Again, CK measurement demonstrated that overexpression of MG53 could ameliorate I/R-induced injury to the myocardium (Supplementary Figure S2D). Cardiac remodeling and contraction analysis also suggested that overexpression of MG53 in blood circulation inhibited cardiac fibrosis and preserved cardiac contractile function in chronic settings (Supplementary Figures S2E–G).

Although MG53 is highly expressed in mouse hearts, its expression level in human hearts remains controversial (24, 25). Cooper's group reported that the human heart lacked MG53 protein, which could not be used as a clinical biomarker of myocardial injury (25). However, studies from our laboratory and other group suggested that MG53 protein could be detected in human hearts (24, 26). To reveal the role of MG53 in the human heart, we used the human-induced pluripotent stem cells (iPSCs) as a model to differentiate into human cardiomyocytes *in vitro*. As shown in Supplementary Figure S3A, although expression of MG53 decreased during the differentiation process, we can detect the expression of MG53 protein in mature beating iPSC-derived cardiomyocytes. To induce injury, we used hypoxia and re-oxygenation (H/R) to mimic *in vivo* I/R injury to the myocardium. Human recombinant MG53 (rhMG53) protein was added to the culture media to test its potential protective effects in iPSC-derived cardiomyocytes. Propidium iodide (PI) is a plasma membrane's impermeable dye and has been widely used to stain cell death events that feature compromised membrane integrity (12, 27). PI staining of H/R-stressed cardiomyocytes indicated that rhMG53 treatment significantly reduced the death of cardiomyocytes (Supplementary Figure S3B). Taken together, our *in vivo* and *in vitro* data suggested that MG53 serves as a protective factor for both mouse and human cardiomyocytes in stressed conditions, which is consistent with the previous studies (7–10).

### ROS Scavenging Counteracts rhMG53 Mediated Cardioprotection

**Figure 1:**
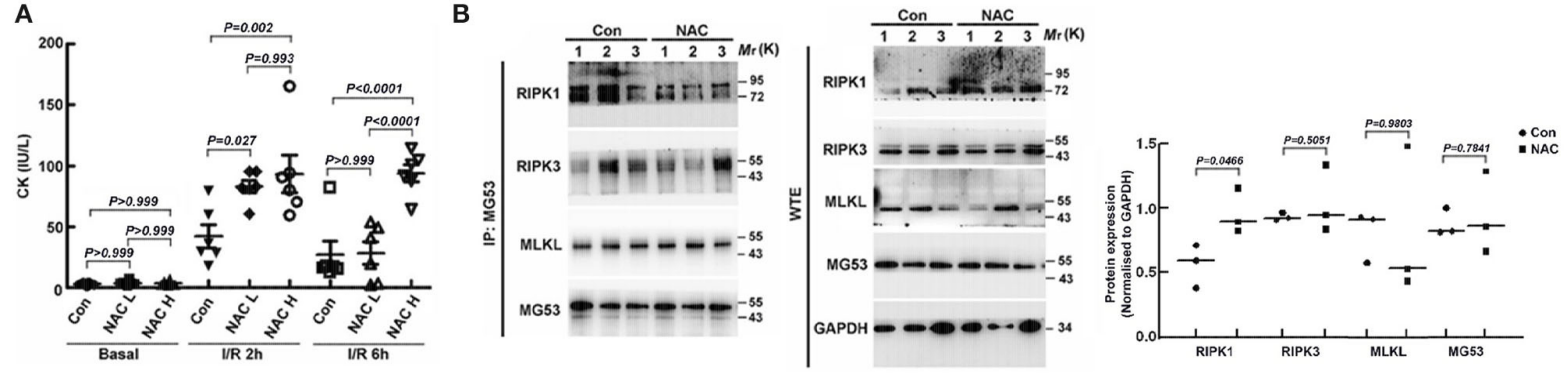
ROS plays a role in regulating the interaction between MG53 and RIPK1 in the injured myocardium. **(A)** The cardiac protective action of rhMG53 was determined by CK lease assay. The beneficial effects of rhMG53 treatment were blunted by treatments of low dose (50 mg/kg, NAC L) and high dose (250 mg/kg, NAC H) of NAC (*n* = 6 per group for control, NAC low dose and NAC high dose). The animals in all experimental groups were treated with rhMG53. **(B)** NAC (50 mg/kg) was injected (IP) into mice before ischemia and reperfusion. Interaction between MG53 and RIPK1 in injured myocardium was determined by co-immunoprecipitation with MG53 antibody and western blot analysis of RIPK1, RIPK3, MLKL, and MG53, the key component of necroptosis complex. (*n* = 3 per group for control and NAC treatment). IP, immunoprecipitation samples; WTE, whole tissue extraction samples.

One of the main consequences of I/R injury is elevation of oxidative stress that compromises cardiac function (26). Since MG53 repair plasma membrane damage associated with I/R injury to the hearts, we hypothesized that combination of rhMG53 with ROS scavenger might play synergistic role in protecting hearts against I/R injury. Thus, we treated mice with N-acetyl-cysteine (NAC), a widely used antioxidant, together with rhMG53 in mice with I/R injury. To our surprise, administration of NAC together with rhMG53 blunted MG53 mediated cardiac protection as demonstrated by CK measurement (Figure 1A). More importantly, counteraction of NAC on MG53 mediated cardiac protection is dose dependent. To determine the potential molecular mechanism underlying this observation, co-immunoprecipitation experiments were performed to determine the potential patterners that interact with MG53 after I/R injury. As shown in Figure 1B, while the level of RIPK1 increased in NAC treated cardiac tissues (WTE in Figure 1B), the RIPK1 that was interacted with MG53 was decreased in NAC treated tissues (IP in Figure 1B). The results suggested that treatment of NAC partially disrupted interaction between MG53 and RIPK1 (Figure 1B), thus, ROS might be a factor to promote MG53/RIPK1 interaction.

### MG53 Protects Against I/R-Induced Cardiac Injury *via* Inhibiting Necroptosis

**Figure 2:**
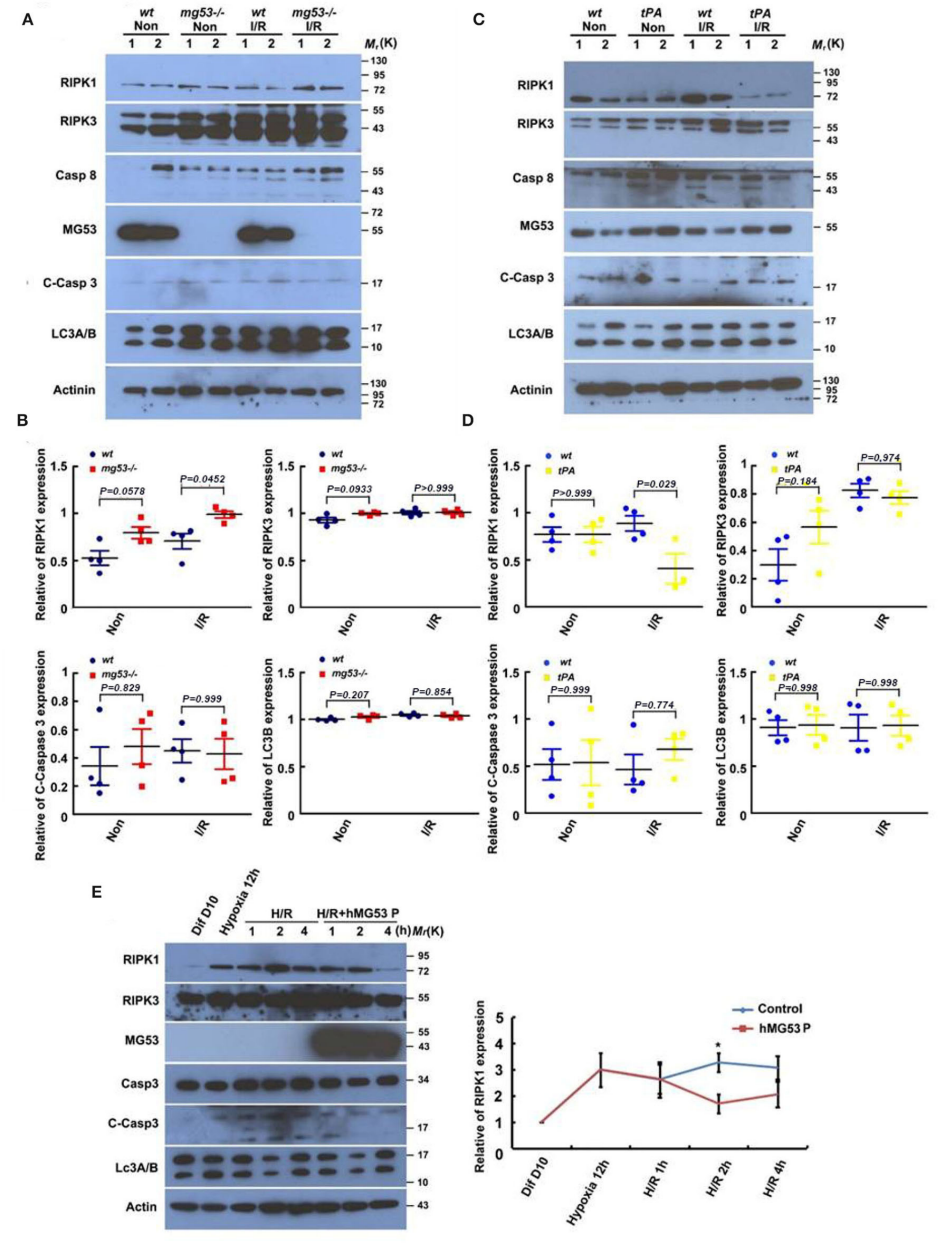
MG53 inhibits necroptosis in cardiac I/R injury *via* regulating RIPK1. **(A)** Western blot analysis of RIPK1, RIPK3, and caspase-8 (necroptosis markers), cleaved caspase-3 (apoptosis marker), and LC-3 II (autophagy marker) in infarct (wt I/R and KO I/R) and non-infarct (wt Non and KO Non) areas of wt and *mg53–/–* hearts. *n* = 4 per group. **(C)** The same set of markers was analyzed by western blot in infarct (wt I/R and tPA I/R) and non-infarct (wt Non and tPA Non) areas of *wt* and tPA-MG53 hearts. *n* = 4 per group. **(B,D)** Western blot results were quantified by ImageJ. **(E)** Representative western blot image of the expression of cell death pathway markers in hiPSC-derived cardiomyocyte at indicated time points of H/R stress with or without rhMG53 protein. *n* = 3 independent experiments. Diff 10 days: cells at basal condition (10 days after differentiation), Hypoxia 12 h: cells in hypoxia for 12 h, H/R 1 h (2 h, 4 h): cells with reperfusion for 1 h (2 h, 4 h).

As it is well known, there are three major types of morphologically distinct cell death: apoptosis, autophagy, and necrosis. RIPK1 is a critical regulator of inflammation and necroptotic cell death (27). To test whether MG53 plays a role in modulating cell death pathways following cardiac injury, we first measured the markers of different cell death pathways in wt and *mg53-/-* hearts subjected to I/R injury. RIPK1, RIPK3, and caspase 8 were used as the markers for necroptosis, cleaved caspase 3 was used for apoptosis, and LC3 II was used for autophagy. Interestingly, among these markers, only RIPK1 was found significantly increased in *mg53-/-* myocardium as compared to that in wt myocardium (Figures 2A,B). More importantly, whereas RIPK1 was increased in infarct myocardium of *wt* and *mg53-/-* hearts, the latter expressed higher RIPK1 than that in injured *wt* hearts (Figures 2A,B). Our results suggested that, first, necroptosis was induced upon I/R-induced cardiac injury and second, MG53 might regulate RIPK1-dependent necroptosis during I/R injury. In addition, we performed the same cell death pathway profiling experiments in tPA-MG53 and their *wt* littermates following cardiac I/R injury. Similarly, we found that the RIPK1 level was significantly decreased in the tPA-MG53 myocardium as compared to that in *wt* littermates (Figures 2C,D). Furthermore, a similar observation was made when we treated human iPSC-derived cardiomyocytes with rhMG53 following H/R injury (Figure 2E). We found that the treatment of rhMG53 led to the downregulation of RIPK1 following H/R injury as compared to BSA treatment as a control. Interestingly, this phenomenon was also confirmed in C2C12 cells, a skeletal muscle cell line. To test the role of MG53, we used CRISPR/Cas9-mediated gene editing to knockout MG53 expression. With this cell model, we were able to perform multiple time point analyses following 12-h hypoxia treatment. As shown in Supplementary Figure S4, in C2C12 wt cells, MG53 expression decreased following re-oxygenation, while the RIPK1 level increased. Interestingly, RIPK1 expression remained very high in *mg53-/-* C2C12 cells through all time points of the H/R process. Thus, our data suggested that MG53 might inhibit necroptosis in I/R-injured hearts through downregulating RIPK1 expression.

### MG53 Serves as a Ubiquitin E3 Ligase to Mediate Degradation of RIPK1

**Figure 3:**
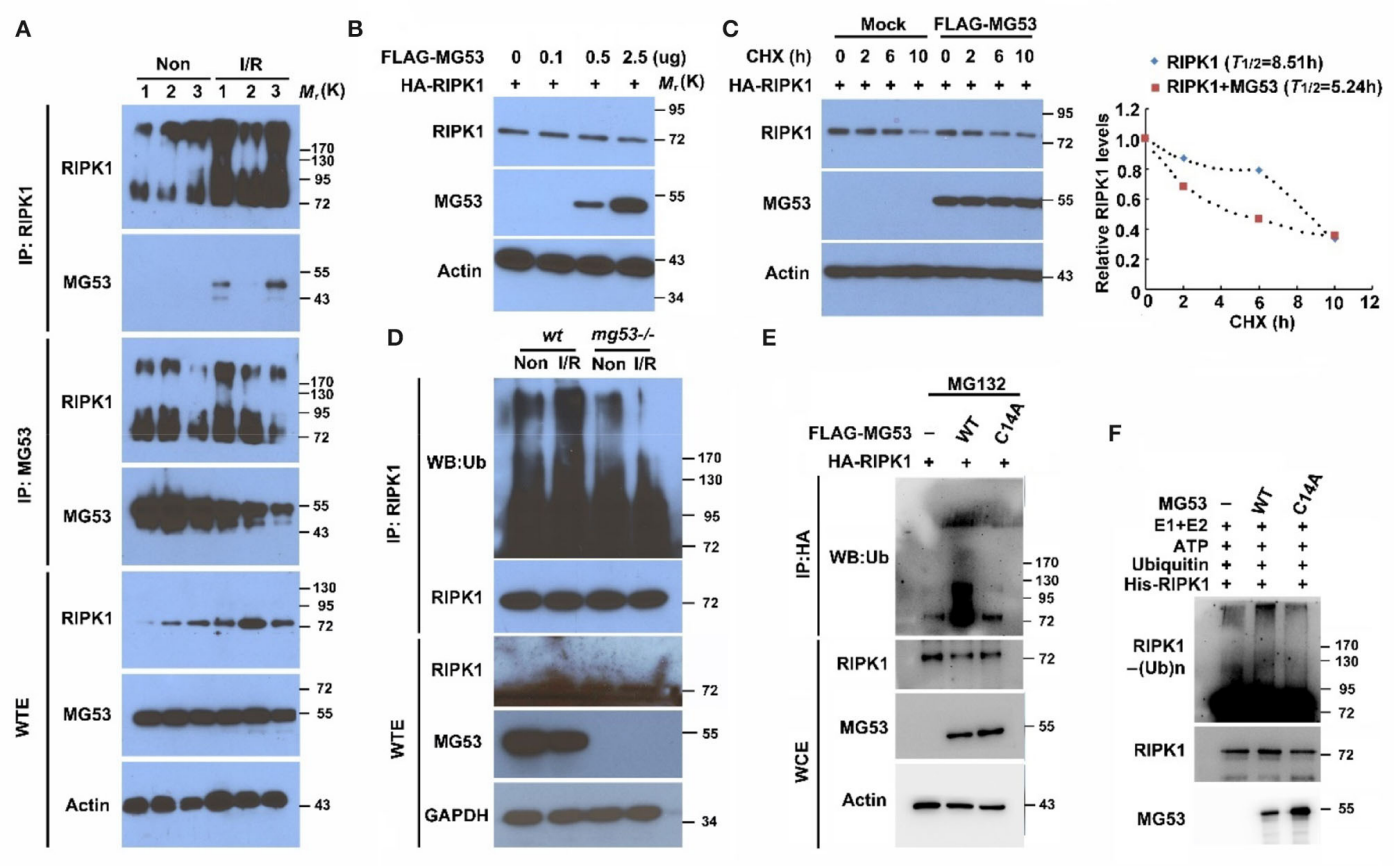
MG53 induces RIPK1 protein degradation through the ubiquitin pathway. **(A)** Co-immunoprecipitation of RIPK1 and MG53 was performed with infarct (I/R) and non-infarct (Non) areas of wt heart lysates. *n* = 3 per group. WTE: whole tissue extraction. **(B)** HEK293 cells were transfected with HA-RIPK1 and different doses of Flag-MG53 plasmid (0–2.5 μg). Western blot analysis showed dose-dependent regulation of RIPK1 by MG53. **(C)** HEK293 cells were transfected by either HA-RIPK1 or a combination of HA-RIPK1 and FLAG-MG53. Cycloheximide (CHX) was added to the culture media, and the expression of RIPK1 was measured at indicated time points. The half-life of RIPK1 was calculated by ImageJ analysis (*right panel*). **(D)** Poly-ubiquitination of RIPK1 was detected in wt and mg53-/- hearts with (I/R) or without (Non) I/R injury. **(E)** HA-RIPK1 was co-expressed either with wt FLAG-MG53 or C14A-MG53 mutant in HEK293 cells. MG132 (10 μM) was used to block protein degradation. **(F)** *In vitro* ubiquitination of RIPK1 was performed with either wt recombinant or mutant (C14A) recombinant MG53 protein. Poly-ubiquitination of RIPK1 was determined by western blot analysis.

To further investigate the relationship between MG53 and RIPK1 during I/R injury, we performed co-immunoprecipitation to measure their direct interaction. We found that RIPK1 antibody could pulldown MG53 protein in I/R-injured myocardium (Figure 3A), suggesting a role of MG53-mediated regulation of RIPK1 during I/R injury. Conversely, the MG53 antibody could pulldown RIPK1 in the injured myocardium (Figure 3A). Interestingly, the level of MG53 protein was decreased in infarct myocardium as compared to remote non-infarct myocardium; however, MG53 antibody could co-immunoprecipitate a similar amount of RIPK1 from both infarct and non-infarct areas, indicating a stronger interaction between MG53 and RIPK1 in injured myocardium (Figure 3A). More importantly, we also observed that the RIPK1 bands had more smear patterns in injured myocardium samples than those in uninjured samples (Figure 3A), suggesting there might be posttranslational modification of RIPK1 following I/R injury. Given the background that MG53 is an E3 ubiquitin ligase (6, 27–29), we hypothesized that MG53 might act as an E3 ligase to downregulate RIPK1 through the protein ubiquitination pathway.

To identify the potential molecular mechanism underlying MG53-mediated RIPK1 degradation, we analyzed this regulation *in vitro* cell system. HEK293 cells were transfected with different MG53 and RIPK1 expression constructs. We found that the expression of RIPK1 decreased as we increased the amount of MG53 expression construct (Figure 3B). This observation was also confirmed in our established C2C12 cell lines, whereas the RIPK1 level was significantly increased in C2C12 *mg53-/-* cells as compared to *wt* parental cells (Supplementary Figure S4). Furthermore, we performed pulse-chase experiment to determine the half-life of RIPK1 protein. We found that overexpression of MG53 significantly accelerated the degradation of RIPK1 (Figure 3C, half-life time of RIPK1 in the presence of MG53: 5.24 h vs. half-life time of RIPK1 in the absence of MG53: 8.53 h), indicating that MG53 might serve as an E3 ligase to regulate RIPK1 protein stability. To test this hypothesis, myocardium derived from *mg53-/-* and *wt* mice was immunoprecipitated with RIPK1 antibody and probed with antibody against ubiquitin. As shown in Figure 3D, ubiquitinated RIPK1 was reduced in *mg53-/-* myocardium, suggesting the presence of MG53-mediated ubiquitination of RIPK1. MG53 belongs to the TRIM family protein which contains the RING domain as a catalytic center for transferring ubiquitin molecules from E2 ligase to its substrates. Previous studies have identified that cysteine 14 is one of the key amino acids to form functional RING structure in MG53 protein, and thus, mutating cysteine 14 to alanine can completely abolish E3 ligase activity of MG53 (30). As shown in Figure 3E, expression of C14A-MG53 produced less poly-ubiquitinated RIPK1 than the expression of wt MG53 did. Since transfection experiments in the cell system might cause false-positive results due to other potential factors present in the cells, we used purified recombinant proteins to perform an *in vitro* ubiquitination assay, as consistent with our observation in the cell culture system, and we found that wt rhMG53 could poly-ubiquitinate recombinant RIPK1 protein *in vitro*, whereas ubiquitinated RIPK1 was significantly reduced when incubated with purified C14A-MG53 protein (Figure 3F). In conclusion, these data suggested that MG53 is an E3 ligase to directly target RIPK1 for its ubiquitination.

### MG53-Mediated Ubiquitination of RIPK1 at Lysines 316, 604, and 627 Sites

**Figure 4:**
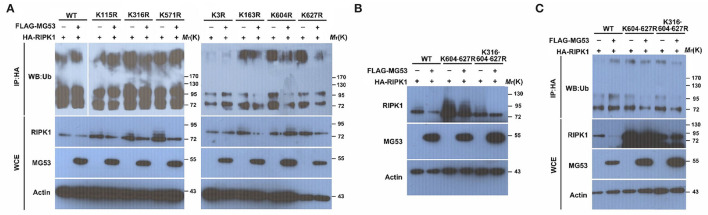
The Lys316, Lys604, and Lys627 residues in RIPK1 are crucial for MG53-mediated ubiquitination. **(A)** wt as well as 7 individual RIPK1 mutant constructs with lysine to arginine mutation were co-transfected with FLAG-MG53 plasmid in HEK293 cells. Ubiquitination of RIPK1 was determined by western blot and compared with or without MG53. **(B)** double (K604-627R) and triple (K316-604-627R) mutation of RIPK1 were co-transfected with or without FLAG-MG53. Levels of RIPK1 were determined by western blot. **(C)** Ubiquitination of wt and mutant RIPK1 was detected with or without FLAG-MG53.

Since lysine residues in the substrate proteins are the targets for ubiquitin modification, we next attempted to identify which lysine residue within RIPK1 is responsible for MG53-mediated poly-ubiquitination. There are 7 lysines in RIPK1 protein, and each individual RIPK1 lysine site was mutated to arginine (29) and tested for MG53-mediated ubiquitination. After the WT and mutant RIPK1 plasmids were co-transfected with MG53 plasmid into the cells, we found that the mutant proteins of K316R, K604R, and K627R were partially resistant to MG53-mediated ubiquitination, suggesting that these three sites are important for the MG53 regulation (Figure 4A). Furthermore, we generated double (K604·627R) and triple (K316·604·627R) RIPK1 mutant constructs. When we co-expressed these mutant constructs with MG53 expression plasmid, we found that both MG53-mediated RIPK1 protein degradation (Figure 4B) and poly-ubiquitination (Figure 4C) were reduced. Taken together, our study showed, for the first time, that ROS generated within injured myocardium could promote interaction between MG53 and RIPK1. MG53 serves as an E3 ligase to ligate ubiquitin to K316, K604, and K627 of RIPK1 and promotes its degradation, which in turn inhibits necroptosis and protects against I/R-induced cardiac injury (Section Graphical Abstract). Thus, functional interaction between MG53 and RIPK1 might be a potential target for treating ischemic heart diseases.

## Discussion

Over the past decade, emerging studies have suggested the existence and significance of necroptosis in the development of heart failure (30–32). Briefly, there are three major complexes in this pathway that determine the cell fate in different conditions. Upon induction, such as TNFα, TNFα receptors can recruit cytosolic adaptor proteins to form complex I, which contains RIPK1 (14, 33). As the process proceeds, the membrane-bound complex I will recruit Fas-associated death domain (FADD) and caspase-8 (Casp8) forming complex II (12, 27). Depending on the activity of Casp8 in complex II, the cells will either undergo apoptosis (active Casp8, complex IIA) or necroptosis (inactive Casp8, complex IIB). In complex IIB, kinase active RIPK1 will phosphorylate and activate RIPK3, and the latter will subsequently phosphorylate mixed lineage kinase domain-like protein (MLKL) to promote its oligomerization and form a nonspecific membrane pore, which disrupts homeostasis of Ca^2+12^ or Na^+12^. Subsequently, necroptosis is executed (6, 29, 34–36). Thus, targeting key molecules in this highly regulated cell death pathway might shed light on our current treatments of myocardial infarction. Here, we showed that MG53 could protect the heart as an E3 ligase to ubiquitinate and degrade RIPK1 to reduce necroptosis during cardiomyocyte I/R injury. Many investigations previously published that MG53 could protect against heart injury in mouse and porcine model (9, 12, 32, 34, 37), however, the role of MG53 as an E3 ligase for tissue protection has not been extensively studied. Our study is the first to describe that MG53 could protect against cardiac I/R injury through ubiquitination-dependent RIPK1 degradation. To our knowledge, previous elegant studies have also identified other E3 ligases of RIPK1 in different physiological and pathophysiological conditions (38–43), which suggested the importance of ubiquitination of RIPK1 in these conditions. Since RIPK1 has 7 lysine residues, the ubiquitination of each residue might be tissue- and disease-specific. Furthermore, not all ubiquitination leads to RIPK1 degradation, for example, Wei et al. have shown that lineage ubiquitination of RIPK1 regulates its kinase activity (39), which adds more levels of regulation of RIPK1 by ubiquitination.

In the present study, we not only found the regulation of RIPK1 specifically in cardiac injury but also indicated a ROS-mediated action on the interaction between MG53 and RIPK1. We found that MG53/RIPK1 interaction requires the presence of ROS; thus, fine-tuning ROS levels in the body might be critical for treating human diseases. As a matter of fact, although ROS has been demonstrated as a major detrimental factor in I/R injury, the clinical trials that use antioxidants to treat cardiovascular diseases usually turn out to be negative (44–47), suggesting that ROS might play some beneficial roles in diseases. In addition, ROS also plays important role in ischemic preconditioning mediated cardiac protection (48–50). Interestingly, previous studies have determined that MG53 plays a critical role in cardiac ischemic preconditioning (8) and post-conditioning (7). Thus, one of our future studies will be focused on dissecting the role of ROS in the regulation of MG53's function in cardiac preconditioning and post-conditioning. In summary, our results might provide a molecular basis for the beneficial role of ROS in protecting against I/R-induced cardiac injury.

In addition, although there was a report showing that MG53 is not expressed in human hearts, suggesting that MG53 might not be required for human heart physiology and diseases, our current results together with previous publications (24, 26) demonstrated the expression of MG53 in human iPSC-derived cardiomyocytes and human hearts. Thus, the complicated regulation of MG53 expression in human hearts might be a future research direction. Furthermore, since MG53 is a cell membrane repair protein, our next studies will focus on testing whether MG53 could repair MLKL-induced plasma membrane injury. Our recent publication did support this hypothesis in liver cells (51); however, more studies are required in cardiomyocytes. Nevertheless, our data support the future clinical application of rhMG53 to treat ischemic heart diseases in human patients.

## Data Availability Statement

The original contributions presented in the study are included in the article/Supplementary Material, further inquiries can be directed to the corresponding author/s.

## Ethics Statement

The animal study was reviewed and approved by IACUC Committee at the Ohio State University.

## Author Contributions

HZ, JM, and QW designed the research. QW, KP, BG, CY, QJ, FY, TT, XZ, and ZB performed research and data analyses. HZ and QW wrote the manuscript. All authors contributed to the article and approved the submitted version.

## Funding

This work was supported by the NIH Grants (AR067766, EY030621, and HL153876) and the AHA Grant (19TPA34850169) to HZ.

## Conflict of Interest

JM and TT had an equity interest in TRIM-edicine, Inc., which develops rhMG53 for treatment of human diseases. Patents on the use of MG53 were held by Rutgers University—Robert Wood Johnson Medical School. The remaining authors declare that the research was conducted in the absence of any commercial or financial relationships that could be construed as a potential conflict of interest.

## Publisher's Note

All claims expressed in this article are solely those of the authors and do not necessarily represent those of their affiliated organizations, or those of the publisher, the editors and the reviewers. Any product that may be evaluated in this article, or claim that may be made by its manufacturer, is not guaranteed or endorsed by the publisher.
