# Supplementary material for: MG53 Inhibits Necroptosis Through Ubiquitination-Dependent RIPK1 Degradation for Cardiac Protection Following Ischemia/Reperfusion Injury
Source: Front Cardiovasc Med. 2022 May 31;9:868632. doi: 10.3389/fcvm.2022.868632 (PMC9193967; doi:10.3389/fcvm.2022.868632)
Supplement: Supplementary file 1 [file Data_Sheet_1.pdf]

Supplemental Figure s1. *Wang et al*

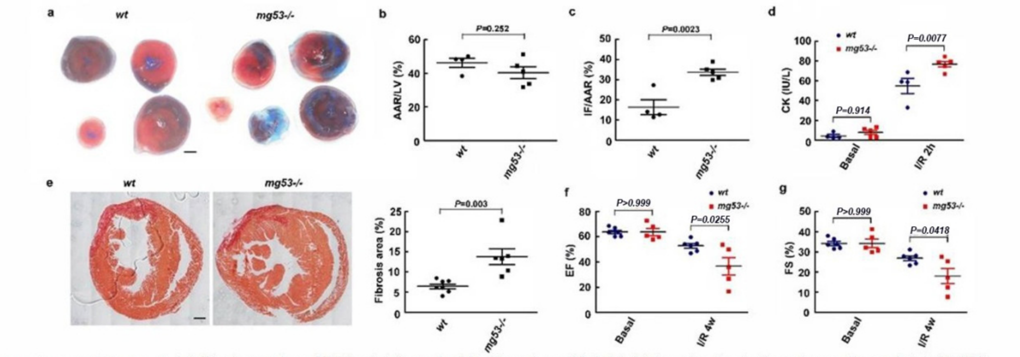

Supplemental Figure s1. (**a**) Photographs of TTC staining of *wt* (*left*) and *mg53-/-* (*right*) hearts. Scale bar: 1mm. Area at risk (AAR)/ left ventricle (LV) ratio (**b**) and infarct size (IF)/ AAR ratio (**c**) of hearts derived from *wt* and *mg53-/-* following I/R injury mice. n=4 for *wt* and 5 for *mg53-/-*. (**d**) Plasma CK level from *wt* and *mg53-/-* mice was measured at indicated time points. n=4 for *wt* and 5 for *mg53-/-*. (**e**) Photographs of Picrosirius red staining of wt (*left*) and *mg53-/-* (*middle*) hearts on 4 weeks after I/R injury. Fibrosis area was quantified (*right*) by ImageJ. n=7 for *wt* and 6 for *mg53-/-*; scale bar, 500 µm. Ejection fraction (EF) (**f**) and fractional shortening (FS) (**g**) of *wt* and *mg53-/-* hearts were measured by echocardiogram. n=7 for *wt* and 6 for *mg53-/-*.

Supplemental Figure s2. *Wang et al*

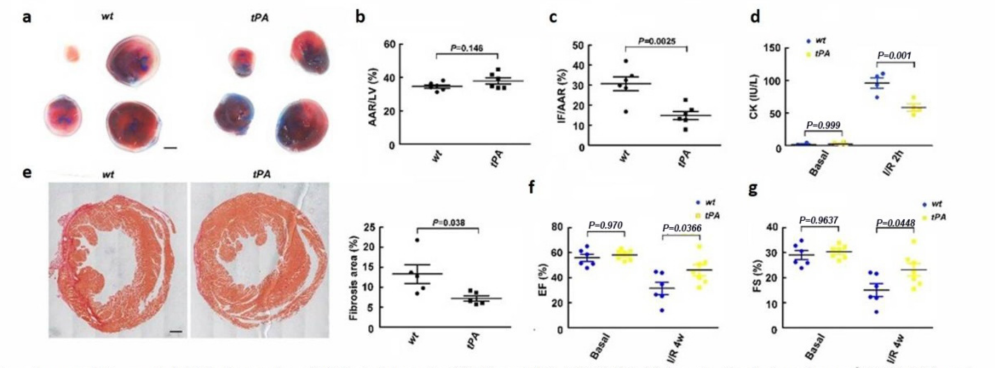

Supplemental Figure s2. (**a**) Photographs of TTC staining of *wt* (*left*) and tPA-MG53 (*right*) hearts. Scale bar: 1mm. AAR/LV (**b**) and IF/AAR (**c**) of wt and tPA-MG53 were quantified. (**d**) CK release in wt and tPA-MG53 was measured at basal and 2 hr after reperfusion, n=6 mice per group. Four week after I/R injury, cardiac fibrosis in *wt* and *tPA-MG53* hearts was determined by Picrosirius red staining (scale bar: 500 μm) and quantified by ImageJ (n=5 per group) (**e**). Echocardiogram was used to determine EF (**f**) and FS (**g**) of wt and tPA-MG53 hearts at basal and 4 weeks after I/R injury (n=6 for *wt* and 7 for tPA-MG53).

Supplemental Figure s3. ***Wang et al***

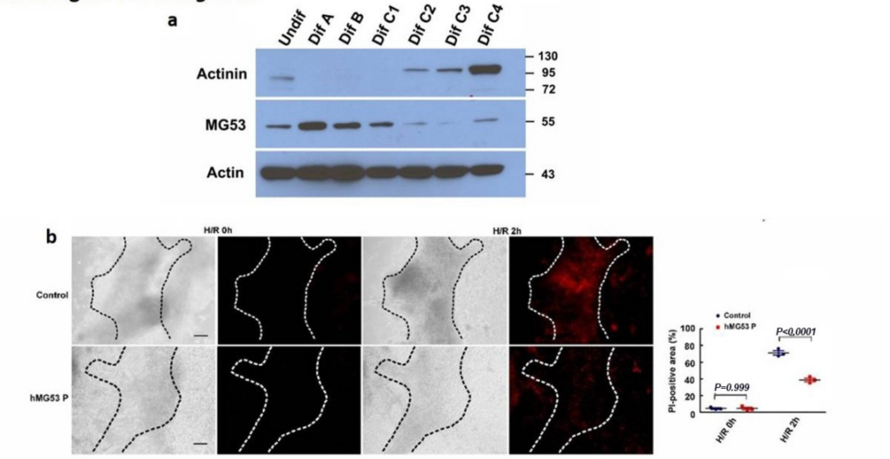

Supplemental figure s3. (**a**) Dynamic expression of MG53 was determined by Western blot. Undif: human iPSC, Dif A: iPSC in differentiation medium A; Dif B: iPSC in differentiation medium B; Dif C1 (C2, C3, and C4): iPSC in differentiation medium C for 1day (2 days, 3 days and 4 days), The beating iPSC derived cardiomyocytes are usually observed on day 3 in differentiation medium C. (**b**) PI staining and quantification of human iPS derived cardiomyocyte following H/R stress with or without rhMG53 treatment. Scale bar, 100 µm. Data are mean ± SEM.

Supplemental Figure s4. *Wang et al*

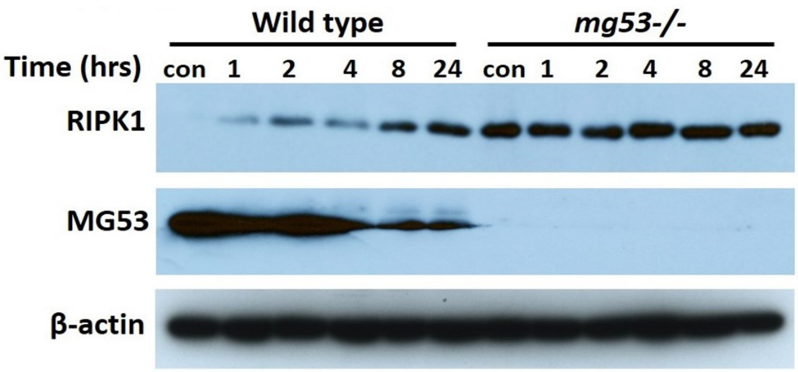

Figure s4 Dynamic expression of RIPK1 and MG53 was determined by Western blot at indicated time points in wt C2C12 cells (wild type) and *mg53-/-* C2C12 cells (*mg53-/-*) following 12 hr hypoxia and reperfusion (1, 2, 4, 8 and 24 hrs). While expression of MG53 declined and RIPK1 expression increased following reperfusion in C2C12 cells, expression of RIPK1 remained high throughout all reperfusion time points in *mg53-/-* C2C12 cells.
